# The complete mitochondrial genome of the thrips predator *Montandoniola moraguesi* (Hemiptera: Anthocoridae) and its phylogenetic analysis

**DOI:** 10.1080/23802359.2026.2713242

**Published:** 2026-08-11

**Authors:** He-Wang Wang, Fu-Ying Guo, Xing Wang

**Affiliations:** a College of Plant Protection, Hunan Provincial Key Laboratory for Biology and Control of Plant Diseases and Insect Pests, Hunan Agricultural University, Changsha, China; b Tropical Biodiversity and Bioresource Utilization Laboratory, Qiongtai Normal University, Haikou, China

**Keywords:** Mitochondrial genome, Anthocoridae, molecular identification, phylogenetic analysis

## Abstract

*Montandoniola moraguesi* (Puton) is a widely deployed biological control agent against gall-inducing thrips, yet genomic resources for its accurate identification and phylogenetic placement remain limited. In this study, we present the complete mitochondrial genome of *M. moraguesi* to characterize its genomic organization and clarify its phylogenetic position within Anthocoridae. The circular mitogenome is 15,062 bp in length, exhibits a high A + T content of 74.04%, and contains the typical set of 37 mitochondrial genes along with a control region. Gene organization follows the ancestral insect mitochondrial pattern. Structural annotation revealed that *COX2* and *ATP6* terminate with incomplete stop codons (T–), while *trnS1* lacks the dihydrouridine (DHU) arm. Phylogenetic analysis based on all 13 protein-coding genes strongly supported *M. moraguesi* as the sister lineage to the genus *Orius* (BS = 100, PP = 1.00). These results provide molecular validation for the morphological classification of the tribe Oriini and supply essential genetic markers for distinguishing this predator from closely related species in integrated pest management strategies.

## Introduction

**Figure 1.**
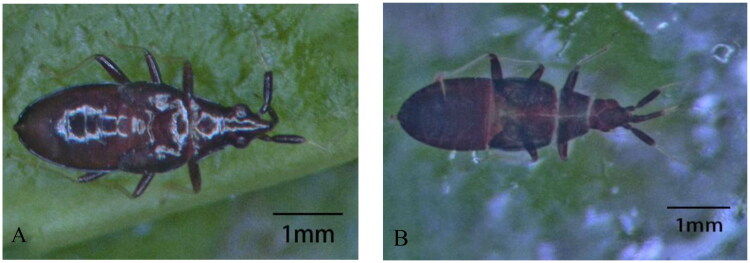
Representative photograph of a nymph of M. moraguesi (Puton, 1896) (sex undetermined). Note: the complete mitogenome was sequenced from a single female adult individual, which was destroyed during total genomic DNA extraction. (A) dorsal view; (B) ventral view. Photograph by Fu-Ying guo.

*Montandoniola moraguesi* (Puton, 1896), an anthocorid predator, is a key natural enemy of the Cuban laurel thrips, *Gynaikothrips ficorum*, throughout tropical and subtropical regions (Carayon and Ramade 1962). This species plays a vital predatory role in the ecosystem, with a documented prey range encompassing more than 20 species of gall-inducing thrips (Sabelis and van Rijn 1997; Dobbs and Boyd 2006). Due to its exceptional efficacy in controlling thrips populations that infest ornamental and economic *Ficus* trees, *M. moraguesi* (Figure 1) has been intentionally introduced to areas such as Hawaii, Bermuda, and the continental United States for biological control practices (Reimer 1988). These introductions have led to significant economic benefits and reduced reliance on chemical pesticides (Shogren and Paine 2016).

Despite its established role in integrated pest management (IPM), genetic studies on *M. moraguesi* remain relatively limited. Compounding this, the species has historically been confused with closely related species, such as *M. confusa* (Pluot-Sigwalt et al. 2009). Given the utility of mitochondrial genomes in resolving insect phylogeny and systematics, we have sequenced, assembled, and analyzed the first complete mitochondrial genome of *M. moraguesi* and reconstructed its phylogenetic relationships based on a multi-gene dataset. This study provides essential molecular data that will aid in the accurate species identification of this important natural enemy, support the conservation of its genetic resources, and promote its further utilization in sustainable pest management strategies.

## Materials and methods

1. Sample Collection and DNA Extraction

Specimens of *Montandoniola moraguesi* were collected manually from the campus of Qiongtai Normal University, Haikou, China (19.9744°N, 110.5167°E). All collected individuals were preserved in anhydrous ethanol, stored at −20 °C, and subsequently deposited as voucher material at Oiongtai Normal University under the accession number QT UDI038.

Total genomic DNA was extracted from the whole body of a single female adult specimen of *M. moraguesi* using the SteadyPure Universal Genomic DNA Extraction Kit Ver.1.0 (Accurate Biology, Changsha, China) following the manufacturer’s instructions. DNA integrity was evaluated by 1% agarose gel electrophoresis.

2. Genome Sequencing, Assembly, and Annotation

High-quality genomic DNA was submitted to Berry Genomics (Beijing, China) for library construction and sequencing on an Illumina NovaSeq 6000 platform, generating 150 bp paired-end reads (PE150).

Adapter sequences and low-quality sequences were removed from the raw sequencing dataset employing Fastp (Chen et al. 2018). The clean data were assembled into a complete mitochondrial genome with GetOrganelle (Jin et al. 2020), and the circularity of the resulting contig was confirmed.

Mitogenome annotation was performed using the MITOS (Bernt et al. 2013). Start/stop codon positions and tRNA gene boundaries were manually verified and adjusted by aligning with homologous sequences from related species in Geneious (Kearse et al. 2012). A circular genome map was visualized using Proksee (Grant et al. 2023).

3. Phylogenetic Analysis

Amino acid sequences of all 13 protein-coding genes (PCGs) were individually extracted and aligned using Geneious Prime v2024 with the MAFFT v7.490 plugin.

Phylogenetic reconstruction was performed using the Maximum Likelihood (ML) method implemented in IQ-TREE v2 (Minh et al. 2020). The best-fit substitution model, GTR+F + I + G4, was selected by ModelFinder (Kalyaanamoorthy et al. 2017). Branch support was evaluated with 1,000 bootstrap replicates.

Additionally, Bayesian Inference (BI) was performed using PhyloSuite v1.2.2 (Zhang et al. 2020). Preliminary tree inspection was conducted in FigTree v1.4.4 (Rambaut 2018), while the final phylogenetic tree was annotated and polished using the Interactive Tree of Life (iTOL) web platform (Letunic and Bork 2024).

## Results

### Mitogenomic architecture

**Figure 2.**
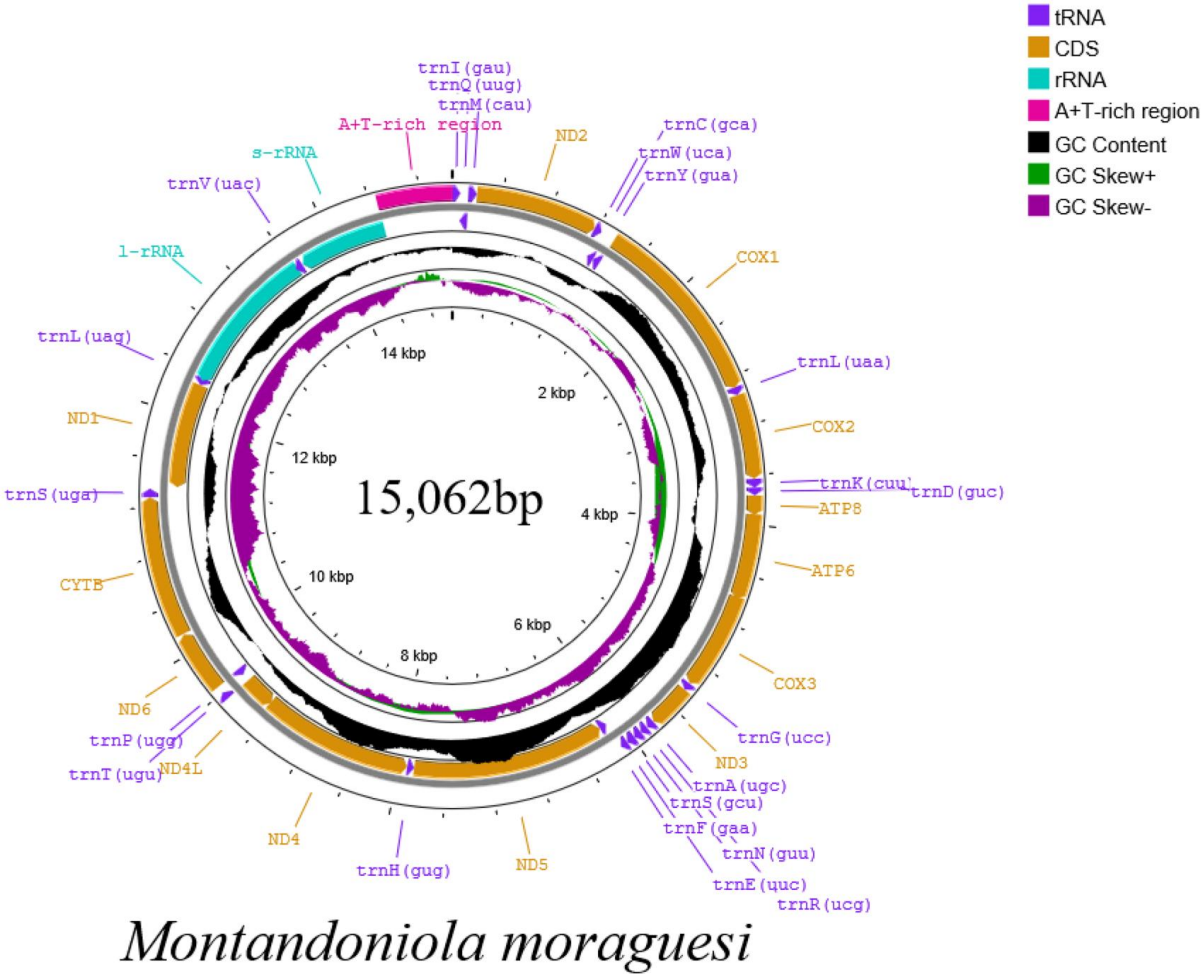
Circular map of the *M. moraguesi* mitochondrial genome. The outer ring represents genes encoded on the majority strand (J-strand), whereas the inner ring depicts genes on the minority strand (N-strand). Transcription direction is marked by a purple arrow.

The complete mitochondrial genome of *M. moraguesi* (GenBank accession no. PX644758) is a closed circular molecule of 15,062 bp, sequenced with an average coverage depth of 267.47× (Figure S1). It contains the typical metazoan set of 37 mitochondrial genes (Figure 2): 13 protein-coding genes (PCGs), 22 tRNA genes, two rRNA genes, and a putative control region (A + T-rich region). Gene arrangement follows the ancestral insect mitochondrial pattern, with 23 genes located on the majority strand (J-strand) and 14 on the minority strand (N-strand). The genome displays a strong nucleotide skew, with an A + T content reaching 74.04% (distribution: *A* = 42.99%, *T* = 31.05%, *G* = 11.16%, *C* = 14.80%).

### Protein-coding genes and RNA profile

Among the 13 PCGs, start codons follow the standard ATN pattern: 6 begin with ATA, 4 with ATG, and 3 with ATT. 11 PCGs employ conventional stop codons (TAA/TAG); whereas COX2 and ATP6 terminate with incomplete stop codons (T—). The 22 tRNA genes vary in length from 59 bp (*trnC*^GCA^) to 71 bp (*trnK*^CUU^), and exhibit typical cloverleaf secondary structures, except for *trnS1* which lacks the dihydrouridine (DHU) arm. The two ribosomal RNA genes, *rrnL* 1303 bp and *rrnS* 779 bp, are located between *trnL*^UGA^ and *trnV*^UAC^, and between *trnV* ^UAC^ and the control region, respectively.

### Phylogenetic reconstruction

**Figure 3.**
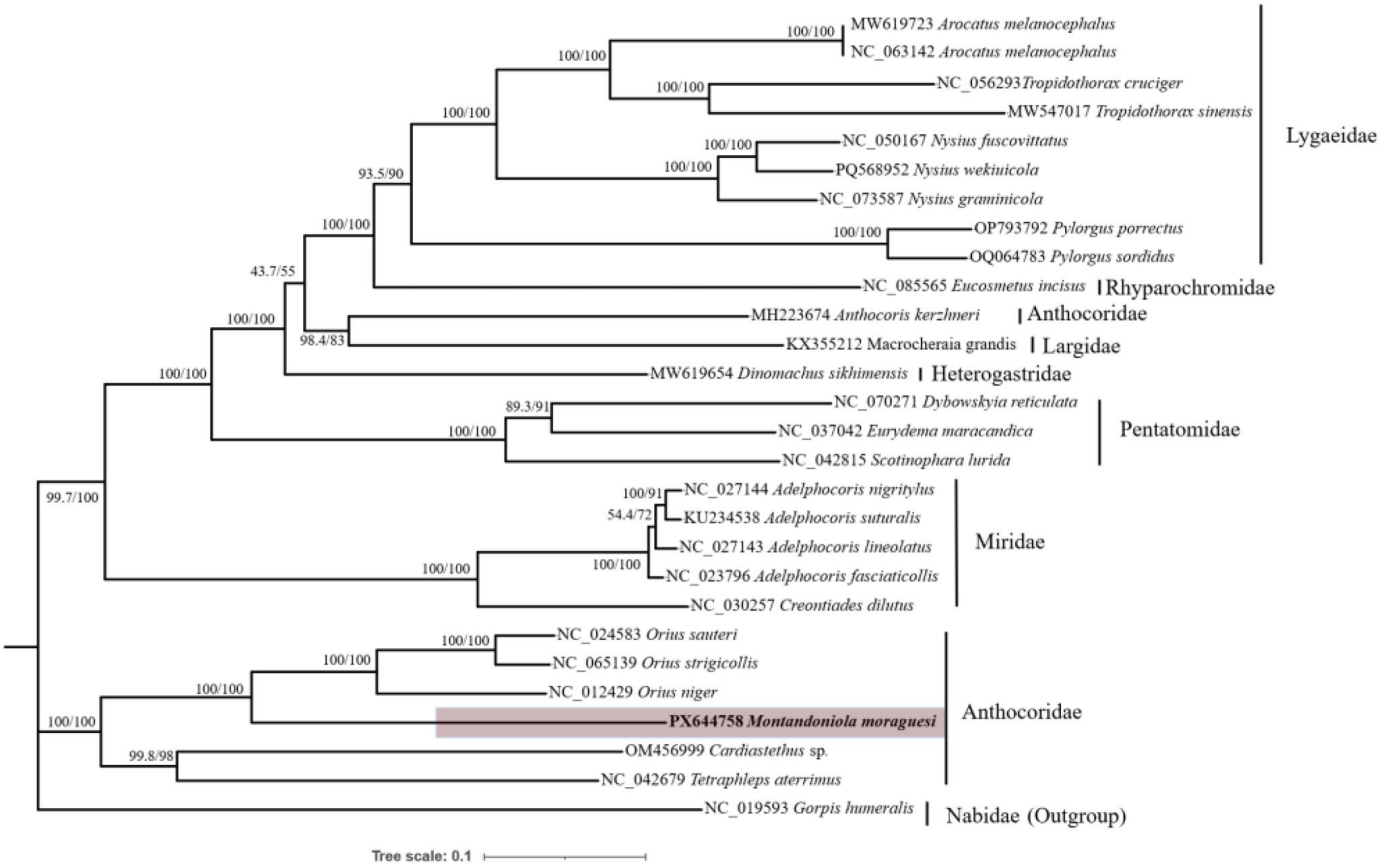
Maximum likelihood (ML) phylogenetic tree of hemiptera species based on complete mitochondrial genomes. GenBank accession numbers are provided alongside the scientific names. Bootstrap support values are indicated at the nodes. *Gorpis humeralis* (nabidae) was designated as the outgroup. Detailed accession information is listed in Table S1. The following sequences were used: MW619723 (Ye et al. 2022b), NC_063142 (Ye et al. 2022c), NC_056293 (Cui et al. 2023), MW547017 (Fei et al. 2021), NC_050167 (Yuan and Cao 2020), PQ568952 (Stever et al. 2024), NC_073587 (Lin and Song 2023), OP793792 (Cao and Dong 2023), OQ064783 (Cao et al. 2023), NC_085565 (Wang 2024), MH223674 (Lin and Song 2019), KX355212 (Liu et al. 2019a), MW619654 (Ye et al. 2022a), NC_037042 (Zhao et al. 2017), NC_070271 (Zhao and Zhao 2023), NC_042815 (Liu et al. 2019b), NC_027144 (Wang et al. 2014b), KU234538 (Zhang et al. 2015), NC_027143 (Wang et al. 2014c), NC_023796 (Wang et al. 2014a), NC_030257 (Hereward 2016), NC_024583 (Du et al. 2014), NC_065139 (Chen 2022), NC_012429 (Hua et al. 2008), PX644758 (present study), OM 456999 (Zhang 2022), NC_042679 (Zhang et al. 2019), and NC_019593 (Li et al. 2012).

Phylogenetic reconstruction using the concatenated nucleotide sequences of all 13 PCGs resulted in congruent topologies between the Maximum Likelihood (ML) and Bayesian Inference (BI) analyses. The resulting phylogenetic tree (Figure 3 and Figure S2) strongly supports the monophyly of the family Anthocoridae. Within this family, *M. moraguesi* was robustly resolved as the sister lineage to the genus *Orius,* with maximal nodal support (BS = 100, PP = 1.00). This clade (*M. moraguesi* + *Orius*) forms a strongly supported sister relationship with the clade comprising *Tetraphleps* and *Cardiastethus*. These results offer molecular confirmation of the placement of *M. moraguesi* within Anthocoridae and clarify its phylogenetic affinities to other anthocorids.

## Discussion

The mitochondrial genome has been proven to be an effective molecular marker for resolving phylogenetic relationships and reconstructing the evolutionary history of Hemiptera (Li et al. 2017). In this study, the phylogenetic tree reconstructed based on all 13 protein-coding genes (PCGs) exhibited extremely high nodal support, reaffirming the reliability of mitogenomic data in resolving taxonomic relationships at the family and genus levels in Heteroptera. Specifically, *Montandoniola moraguesi* clustered with the genus *Orius* with maximal support, forming a sister group. This result is congruent with the morphological classification of Schuh and Slater (1995), providing molecular evidence to substantiate the placement of these genera within the tribe Oriini.

Both *Montandoniola* and *Orius* are recognized as important predators of thrips and other pests in agroecosystems (Arthurs et al. 2011). The phylogenetic relationship confirmed here provides a necessary genetic background for future comparative studies on their evolutionary history.

From an applied perspective, the sequenced mitogenome serves as a basic genetic resource for species identification. Since *M. moraguesi* can be difficult to distinguish from morphologically similar species, such as *M. confusa*, this data provides a critical comparative baseline. Although the complete mitogenome of *M. confusa* is currently unavailable, future studies should prioritize decoding its mitogenome to conduct comparative analyses with our data. This will facilitate the development of robust molecular diagnostic markers for accurate identification. This is particularly valuable for monitoring biological control agents and ensuring the correct species are used in pest management programs.

## Supplementary Material

Supplemental Material

## Data Availability

The genome sequence data supporting the results of this study are openly available in GenBank at NCBI under accession no. PX644758. The associated BioProject, BioSample, and SRA numbers are PRJNA1371101, SAMN53489887, and SRR36238800, respectively.
