# Supplementary material for: The complete mitochondrial genome of the thrips predator Montandoniola moraguesi (Hemiptera: Anthocoridae) and its phylogenetic analysis
Source: Mitochondrial DNA B Resour. 2026 Aug 11;11(9):1019–23. doi: 10.1080/23802359.2026.2713242 (PMC13463533; doi:10.1080/23802359.2026.2713242)
Supplement: Supplementary file 1 [file TMDN_A_2713242_SM4550.docx]

**Supplemental material**

Figure S1. The coverage depth of the mitochondrial genome of *Montandoniola moraguesi.*

Figure S2. Bayesian Inference (BI) phylogenetic tree of Hemiptera species based on complete mitochondrial genomes. GenBank accession numbers are provided alongside the scientific names. Posterior probability values are indicated at the nodes. *Gorpis humeralis* (Nabidae) was designated as the outgroup. Detailed accession information is listed in Table S1.

| Family | Species | Accession Number | Reference |
| --- | --- | --- | --- |
| Lygaeidae | *Nysius fuscovittatus* | NC_050167 | Yuan et al. 2020 |
| Lygaeidae | *Nysius wekiuicola* | PQ 568952 | Stever et al. 2024 |
| Lygaeidae | *Nysius graminicola* | NC_073587 | Lin et al. 2023 |
| Lygaeidae | *Arocatus melanocephalus* | MW619723 | Ye et al. 2022 |
| Lygaeidae | *Arocatus melanocephalus* | NC_063142 | Ye et al. 2023 |
| Lygaeidae | *Tropidothorax cruciger* | NC_056293 | Gui et al. 2023 |
| Lygaeidae | *Tropidothorax sinensis* | MW547017 | Fei et al. 2021 |
| Lygaeidae | *Pylorgus porrectus* | OP793792 | Cao et al. 2023 |
| Lygaeidae | *Pylorgus sordidus* | OQ064783 | Cao et al. 2023 |
| Lygaeidae | *Macrocheraia grandis* | KX 355212 | Liu et al. 2019 |
| Anthocoridae | *Anthocoris kerzhneri* | MH223674 | Li et al. 2019 |
| Heterogastridae | *Dinomachus sikhimensis* | MW 619654 | Ye et al. 2022 |
| Rhyparochromidae | *Eucosmetus incisus* | NC_085565 | Wang 2024 |
| Pentatomidae | *Euryrema maracandica* | NC_037042 | Zhao et al. 2017 |
| Pentatomidae | *Dybowskyia reticulata* | NC_070271 | Zhao et al. 2023 |
| Pentatomidae | *Scotinophara lurida* | NC_042815 | Liu et al. 2019 |
| Miridae | *Adelphocoris suturalis* | KU 234538 | Zhang et al. 2015 |
| Miridae | *Adelphocoris nigritulus* | NC_027144 | Wang et al. 2014 |
| Miridae | *Adelphocoris lineolatus* | NC_027143 | Wang et al. 2014 |
| Miridae | *Adelphocoris fasciaticollis* | NC_023796 | Wang et al. 2014 |
| Miridae | *Creontiades dilutus* | NC_030257 | Hereward 2016 |
| Anthocoridae | *Orius sauteri* | NC_024583 | Du et al. 2014 |
| Anthocoridae | *Orius strigicollis* | NC_065139 | Chen 2022 |
| Anthocoridae | *Orius niger* | NC_012429 | Hua et al. 2008 |
| Anthocoridae | *Montandoniola moraguesi* | PX 644758 | Present study |
| Anthocoridae | *Tetraphleps aterrimus* | NC_042679 | Zhang et al. 2019 |
| Anthocoridae | *Cardiastethus* sp. | OM 456999 | Zhang et al. 2022 |
| Nabidae | *Gorpis humeralis* | NC_019593 | Li et al. 2012 |

Table S1. Summary of species information and GenBank accession numbers used for phylogenetic analysis.
